# Metabotype Dynamics of Market-Ripe Orange (*Citrus sinensis*) During Postharvest Ageing at Room Temperature

**DOI:** 10.3390/metabo16100735

**Published:** 2026-09-30

**Authors:** Mabel M. Montenegro-Sustaita, José Iván Serrano-Contreras, Gabriela Torres-Santiago, Luis Gerardo Zepeda-Vallejo

**Affiliations:** 1 Departamento de Química Orgánica, Escuela Nacional de Ciencias Biológicas, Instituto Politécnico Nacional, Prolongación de Carpio y Plan de Ayala s/n, Col. Santo Tomas, Delegación Miguel Hidalgo, Ciudad de Mexico 11340, Mexico; mmontenegro@ipn.mx; 2 Department of Metabolism, Digestion and Reproduction, Division of Digestive Diseases, Section of Nutrition, Faculty of Medicine, Imperial College London, London W12 0NN, UK; 3 Facultad de Ciencias Químicas, Circuito Universitario s/n, Campus Uach II, Chihuahua, Chih. 31125, Mexico; gsantiago@uach.mx

**Keywords:** *Citrus sinensis*, advanced-ageing metabotype, metabotype complexity, entropy, NMR

## Abstract

**Objectives**: To develop and apply a robust method using NMR-based metabolic phenotyping to characterise the metabolic changes occurring in commercially acquired, market-ripe orange (*Citrus sinensis*—a non-climacteric fruit) during postharvest ageing. **Methods**: Orange juice was obtained at different postharvest timepoints, based on morphological changes in the fruit (e.g., days 9, 30, and 35). The fruits were stored at room temperature (26 °C) and 60% relative humidity, throughout the study. The NMR spectra of the juice were collected and analysed using a semi-targeted approach alongside multivariate, correlation, and entropy analyses. **Results**: A robust method to assess the orange juice metabotype was developed with a total of 42 metabolites, of which two were unknowns, including phenolic compounds, carbohydrates, amino acids (essential, conditionally essential and non-essential), alcohols, and other metabolites that may have direct nutritional value. This method proved to be useful to monitor the progressive metabotype complexity derived from postharvest deterioration. Based on metabolite patterns across postharvest stages, correlation structures, and entropy values, the ageing and advanced-ageing metabotypes were defined at days 30 and 35, respectively. **Conclusions**: Phlorin, galacturonate, succinate, rhamnose and prolinebetaine were associated with ageing profiles, providing a basis for their potential use in targeted monitoring of orange postharvest ageing. These findings could support the future development of compact, affordable devices for rapid in situ assessment of postharvest ageing status, with potential benefits of supporting the preservation of product quality, nutritional and economic values.

## 1. Introduction

Fruits are mature ovaries that have undergone major evolutionary adaptations to improve seed protection and dispersal [1]. Fruit development comprises four stages: (i) fruit set, which includes ovary development and the onset of cell division; (ii) cell division or growth, (iii) cell expansion or maturation, during which the fruit increases mainly in size, and ripening (iv) [2]. Ripening involves a series of coordinated physicochemical changes that enhance the fruit’s nutritional value by establishing an appropriate balance of sugars, organic acids, amino acids, and carotenoids [3,4,5,6]. These changes also improve visual appeal and palatability for consumers [7]. In this context, fleshy fruits are classified as climacteric or non-climacteric according to the differences in respiration and ethylene biosynthesis during ripening. Although these mechanisms have been studied extensively in climacteric fruits, they remain less well understood in non-climacteric fruits such as oranges [6,8,9].

Fruit quality depends on both nutritional composition and organoleptic attributes, which are largely determined by the balance of organic acids, sugars, and other metabolites accumulated during fruit development and present at harvest [2]. These characteristics are influenced by multiple pre-harvest factors that include intrinsic biological factors (e.g., genetic background), abiotic (e.g., cultivation practices, harvest methods, irrigation, mechanical stress), and biotic (e.g., microbial invasion, insect pests). Preharvest stresses can induce physiological and metabolic changes that persist after harvest, thereby influencing fruit quality during postharvest stage [10]. Although the relative impact of each factor is not always easy to define, proper crop management is essential to preserve quality through storage. Once displayed at the point of sale, the shelf life of fruit in general can be affected by postharvest factors including temperature, handling, packaging, relative humidity, physical damage, exposure to pathogens, and chemical treatments, all of which contribute to the fruit quality after harvest [11].

NMR-based metabolic phenotyping is one of the most effective approaches for obtaining a detailed view of the biochemical processes occurring in biological systems [10,12]. NMR is widely used in metabolomics as it offers high reproducibility, enables the simultaneous analysis of metabolites in complex mixtures [13], and facilitates the integration of spectral data with statistical models [14]. Food metabolomics (within the broader field of Foodomics) has been widely applied and is emerging as a valuable long-term strategy for ensuring food quality. It has the potential to have highly positive economic impact, as it involves using analytical developments to protect both producers and consumers [13,15].

Previous ^1^H NMR studies have examined the metabotype of citrus fruits, quantified citrate and malate, and evaluated the effect of temperature on orange juice [16]. A notable study investigated the ageing of the peel of *Citrus reticulata*, a Chinese citrus variety used in traditional medicine, where longer ripening was associated with potential health-related benefits [17]. Additional studies have described the proteomic profile and genetic regulation of *Citrus* sp. as well as the quality control and authenticity of orange juices [18,19]. More recently, NMR-based metabolomics has been used to monitor metabolic changes in Navel orange juice, albedo, and flavedo during long-term cold storage [16]. Therefore, this study aimed to characterise the physiological changes occurring during postharvest ageing in *Citrus sinensis* by monitoring its juice metabotype using NMR-based metabolic phenotyping approach.

## 2. Materials and Methods

### 2.1. Fruits Source

Fresh Valencia oranges (*C. sinensis*) were purchased from a local commercial distributor (Central de Abastos, Tultitlán, Mexico; 19.6397816, −99.1391827) on the 8 May 2019. According to the supplier specifications, the fruits were harvested two days prior to warehouse arrival. However, detailed pre-harvest conditions (orchard microclimate, agricultural management) and postharvest chemical treatments were unrecorded due to commercial supply chain constraints. The fruits were immediately transported to the laboratory, and a total of 70 fruits were selected based on similar size, firm texture, uniform peel colour, and an intact, glossy peel, apparent maturity, and with no visible mechanical damage or deterioration were randomly selected. Then, the fruits were washed with distilled water, gently wiped dry, and randomly allocated into four sampling groups, each corresponding to a defined sampling timepoint. The fruits were stored under controlled conditions at 26 °C and 60% relative humidity until their respective sampling and extraction times.

### 2.2. Sample Preparation

For this study, the day of purchase was defined as the experimental day 0, representing the beginning of the controlled laboratory storage period rather than the harvest date.

To focus specifically on postharvest ageing, the sampling timepoints were defined based on the progressive physical deterioration of the oranges during storage, with the final timepoint corresponding to the appearance of clear signs of rotting.

The selected 70 fruits were randomly allocated to four sampling groups: D9 (n = 20), D18 (n = 10), D30 (n = 20), and D35 (n = 20). However, as the study progressed, eight fruits were excluded when, during sample preparation, they were found to exhibit deterioration that was markedly more advanced or otherwise atypical relative to the other fruits in their respective groups. These atypical features included localised tissue softening, loss of peel gloss and firmness, and the development of brown spots on the peel. The cause of the atypical deterioration could not be established as detailed information on preharvest conditions, handling, transportation, and postharvest treatments was unavailable, as mentioned above. These changes cannot therefore be attributed specifically to postharvest ageing, and then the final sample size comprised 62 oranges.

The first juice extraction was performed on the 17 May 2019, after nine days (D9, n = 17) of controlled storage and approximately 11 days postharvest. Subsequent sampling and juice extraction were conducted on the 7 June 2019, after 30 days (D30, n = 18) of controlled storage and approximately 32 days after harvest; and on the 12th of June 2019, after 35 days (D35, n = 19) of controlled storage and approximately 37 days after harvest. On D35, the oranges exhibited clear visible signs of deterioration. For sensitivity analysis, an intermediate timepoint was introduced by subdividing the interval between D9 and D30, corresponding to Day 18 (D18, n = 8), where sampling was carried out on the 26 May 2019, after 18 days of controlled storage and approximately 20 days after harvest.

For juice extraction, the oranges were peeled, and the juice was obtained using a domestic electric juicer (brand RCA, Mexico branch), which was rinsed with distilled water between each sample to prevent cross-contamination.

The juice obtained from each orange was filtered with acrodisc hydrophilic PES Membrane syringe filter, 25 mm, 0.45 µm (Corning^®^ syringe filters, Kennebunk, ME, USA), 10 mL of the filtrate was taken and centrifuged three times at 3250× *g* and 4 °C for 15 min. The sediment (pulp) was removed between cycles, and 1 M NaOH was added to the clear supernatant until pH 7.0 was reached. Subsequently, the clear supernatant was diluted 1:1 by adding 10 mL of 200 mM phosphate buffer (KH_2_PO_4_: K_2_HPO_4_, 5:3 molar ratio; pH 7.0, 0.1% (*w*/*v*) of sodium azide (99.7%, Sigma Aldrich, Milwaukee, WI, USA)) was added to each sample to maintain pH 7.0. The samples were stored at −78 °C prior to NMR analysis. For NMR analysis, 540 µL of the clear supernatant was transferred to a 5 mm NMR tube and mixed with 60 µL of TSP solution at a concentration of 11 mM in 100% deuterium oxide (D2O), resulting in a final TSP concentration of 1.1 mM and in a total sample dilution factor of 2.2. TSP was used only for chemical-shift referencing.

### 2.3. ^1^H NMR Spectroscopy

Water-suppressed 1D ^1^H NMR spectroscopy was performed at 298 K on a 600 MHz Bruker Avance III spectrometer (Bruker Biospin, Karlsruhe, Germany). The 1D ^1^H NMR spectra were acquired using standard one-dimensional pulse sequence, with saturation of the water resonance (noesypr1d, standard Bruker pulse program) during both the relaxation delay (RD = 4 s) and mixing time (tm = 10 ms). The receiver gain was set to 45.2 and acquisition time (ACQ) to 2.59 s for all experiments. Each spectrum was acquired using 4 dummy scans, 32 scans, 64K data points and with a spectral window of 21 ppm. Prior to Fourier Transformation, each free induction decay was multiplied by an exponential function corresponding to a line broadening of 0.3 Hz. A zero-filling by a factor of 2 was used to produce 128K data points, the first-order phase correction was set to 0.0 and only zero-order phase correction was implemented. For quality assurance, the symmetry of the TSP signal with a line width at half height of ≤1 Hz, and a flat baseline were assessed.

NMR spectra were acquired in batches corresponding to the predefined postharvest ageing timepoints, with the actual acquisition sequence being D9, followed by D18, D35, and finally D30. A total of 62 biological samples were analysed (D9 = 17, D18 = 8, D30 = 18, and D35 = 19).

Homo- and heteronuclear 2D NMR experiments, such as *J*-Resolved, COSY, TOCSY, HMBC and HSQC, were acquired for representative samples to support the spectral assignment.

### 2.4. NMR Data Pre-Processing

All 1D ^1^H NMR spectra were automatically phased, baseline-corrected, and referenced to the singlet of the internal standard (TSP) at δ 0.0 using the Topspin 3.6.1 software (BrukerBioSpin, Rheinstetten, Germany). Full resolution 1D ^1^H NMR spectra (~9K spectral variables) were automatically phased and baseline corrected and then digitised over the range δ −0.5 to 11 and imported into MATLAB (2014a, MathWorks, Natick, MA, USA). The spectral regions corresponding to the TSP (δ −0.5 to 0.7), water (δ 4.7 to 5.2) and noise (δ 9.8 to 11.00) were excluded. Prior to multivariate data analysis, the spectra were normalised using the probabilistic quotient method (PQN) [20].

### 2.5. Identification of Metabolites

Subset optimisation by reference matching (STORM) was used to identify metabolites using the correlation structure of 1D ^1^H NMR data set [21]. Strong coupling artefacts from metabolites produced in the 2D *J*-Resolved spectrum were also used for metabolite identification purposes [22].

Internal and external databases such as the Human Metabolome Data Base (HMDB; http://hmdb.ca/, accessed on 16 January 2026), the Food Database (FOODB; https://foodb.ca/, accessed on 16 January 2026), the Biological Magnetic Resonance Data Bank (BMRB; http://www.bmrb.wisc.edu), nmrshiftdb2 (NMRShiftDB project; https://nmrshiftdb.nmr.uni-koeln.de/, accessed on 16 January 2026), and the database of the Chenomx^®^ NMR suite 8.0.1 were used for confirmation of assignments. The literature was also consulted for identification purposes [23,24,25,26,27,28,29,30].

### 2.6. Semitargeted Approach

The integration of a respective NMR spectral region corresponding to a representative resonance for each identified metabolite was obtained using the following equation:

$$I=\sum_{k=1}^{n}\left(\int_{j_{k}^{h}}^{j_{k}^{i}}I(x)dx\right)$$

where *k* corresponds to a spectral region peak, *I* is the area under the curve of the intensities *I*(*x*) that define a metabolite’s resonance signal, with the spectral region ranging from high-field to low-field ($j_{k}^{h}$ and $j_{k}^{i}$, respectively). A total of 40 metabolites were identified, of which 4 were putatively annotated as phenolic compounds, and 2 were unknown metabolites. This dataset was used to construct the semitargeted matrix (X), with *m* variables (columns) and *n* observations (rows).

To address overlapping and shifted resonances, all spectra were subjected to extensive and careful manual inspection and peak picking. Overlapping resonances were carefully assessed using diagnostic chemical shifts, signal multiplicities, resonance patterns, and comparison with reference spectra acquired from authentic reference standards or from those reported in external databases and/or the literature. For each identified metabolite, a single representative peak was selected, and its area was estimated by trapezoidal numerical integration, using consistently defined spectral regions across all samples to ensure comparable integration despite peak overlap and chemical-shift variation. Where substantial overlap could compromise reliable assignment, only well-resolved resonances were considered for area integration. Using this approach, 42 metabolites were assessed.

### 2.7. NMR Statistical Analysis

The semi-targeted dataset was modelled using orthogonal projection to latent structures discriminant analysis (OPLS-DA) in a Monte-Carlo Cross-Validation (MCCV) framework based on pairwise comparisons. Under MCCV approach NMR dataset is split into many different training and test sets, and each one is auto-scaled using the mean and the standard deviation of the training set, to obtain robust and reliable estimates of the regression coefficients and predicted scores. For each of 100 models, 1/5th of the data is left out and the variability of the regression coefficients is calculated by running an additional 25 models with bootstrap resampling from the training data. These 100 models give a robust estimate of the mean regression coefficients and the 2500 bootstrap models are used to estimate the variance. For each variable, a *t*-score and a *p*-value are calculated using the variance and the mean regression coefficient. The goodness of fit (R^2^Y) of the MCCV models is calculated across all models using the training data and the goodness of prediction (Q^2^Y) for the test data. Permutation testing was not performed within this framework.

The variable significance is represented as “direction of change” (DoC) value:

$${DoC}_{i}=-{log}_{10}(p)\times (\beta_{i}/\left|\beta_{i}\right|)$$

where *βi* is the ith regression coefficient and *p* the *p*-value. Significant changes were identified using Bonferroni correction with a threshold of *p* < 0.05/42 [31]. The MCCV-OPLS-DA code can be accessed through the original developer, as cited in Reference [31].

### 2.8. Correlation Analysis

Metabolite-metabolite correlation analysis was carried out using the Spearman method (rho), with analysis performed separately for each timepoint. The same set of variables was included at each timepoint (n = 42), with no missing values. Thus, all pairwise correlations within a given timepoint were based on the same sample size, with differences in sample size occurring only across timepoints. Statistical significance was assessed using the false discovery rate (FDR) approach implemented in the R package qvalue, where *q*-values were calculated from the resulting two-sided Spearman correlation *p*-values, with the estimation of the proportion of true null hypotheses performed using 5000 bootstrap replicates. A cut off of |*rho*| ≥ 0.75 with *q*-value < 0.05 was used to indicate a significant correlation and to be considered sufficiently strong for inclusion in the final network.

In addition, to assess correlation stability, non-parametric bootstrap resampling with replacement was performed separately for each pairwise association using 5000 bootstrap samples. Each bootstrap sample contained the same number of observations as the corresponding pairwise complete dataset. Spearman correlation coefficients were recalculated for each bootstrap sample, and 95% confidence intervals were obtained using the percentile method.

Network density was also calculated as the number of retained associations divided by the total number of possible pairwise associations.

At each timepoint, the same 42 metabolites were included, resulting in 861 unique pairwise correlations. Thus, the same metabolites and pairwise comparisons were evaluated at each timepoint, with FDR correction applied separately to the 861 tests performed at each timepoint.

### 2.9. Entropy Analysis

Shannon entropy (*H*, also known as raw entropy) was calculated using the correlation matrix per timepoint as follows:

$$H=\;-\;\sum_{i=1}^{N}p_{i}{log}_{2}(p_{i})$$

$$p_{i}=\frac{x_{i}}{\sum_{i=1}^{N}x_{i}}$$

where *p_i_* = normalised probability of event *i* (i.e., normalised correlation magnitude), *x* = vector of correlation magnitudes (absolute values; dividend is the sum of all correlations), *N* = number of correlations (upper triangle of the correlation matrix built per timepoint).

In addition, and for better comparison, normalised entropy was also calculated. First, theoretical maximum entropy (*H_max_*) was computed. Given that *H_max_* for any vector of length *N* does not depend on the actual correlation values, but only on the number of the elements per timepoint [32,33]:

$$H_{max}=\;{log}_{2}(N)$$

Then, normalised entropy (*H_norm_*) is given by:

$$H_{norm}=\frac{H}{H_{max}}$$

For better visualisation, normalised deviation was calculated:

$$Normalised\;deviation=\;1-\;H_{norm}$$

Entropy was calculated from only statistically significant correlations.

Statistical, correlation and entropy analyses as well as data visualisation were performed using MATLAB (2014a, MathWorks, Natick, MA, USA) and R (4.0.0) in RStudio.

The scripts used for the correlation and entropy analyses are available from the corresponding author upon reasonable request.

## 3. Results

**Figure 1:**
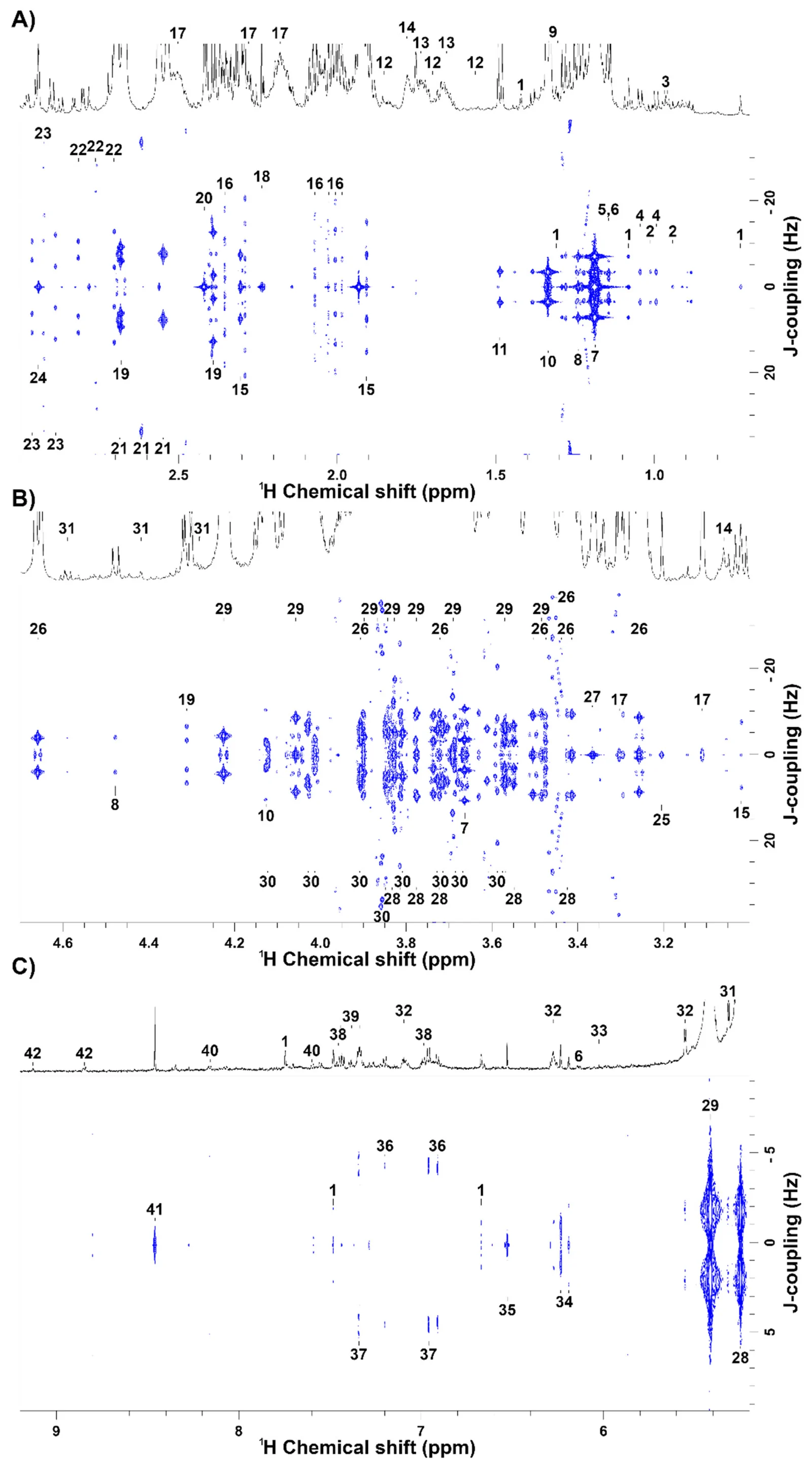
2D *J*-Res spectrum (600 MHz) of market-ripe orange (*C. sinensis*) juice with the 1D ^1^H spectrum plotted above showing the aliphatic (**A**), carbohydrate (**B**) and aromatic (**C**) regions. Detailed metabolite names are provided in Table S1.

The present study describes a comprehensive analysis of *Citrus sinensis* through NMR-based metabolic phenotyping to monitor its physiological changes during postharvest storage. A total of 42 metabolites were assessed, of which 40 metabolites were identified, including four that were putatively annotated as phenolic compounds, while two were unknown metabolites (Figure 1 and Table S1).

Metabolite identification was performed using a combination of spectral database matching and comparison with the published literature. *N*-methyl-Proline and phlorin were assigned based on spectral database matches and the literature evidence; therefore, these metabolites were putatively annotated compounds. Phenolic compounds 1–4 were putatively annotated at the phenolic compound class level based on similarities in spectral fingerprints from known phenolic compounds. The remaining 36 metabolites were confidently identified compounds using spectra acquired from authentic reference standards, spectral database matches, and/or the literature evidence.

**Figure 2:**
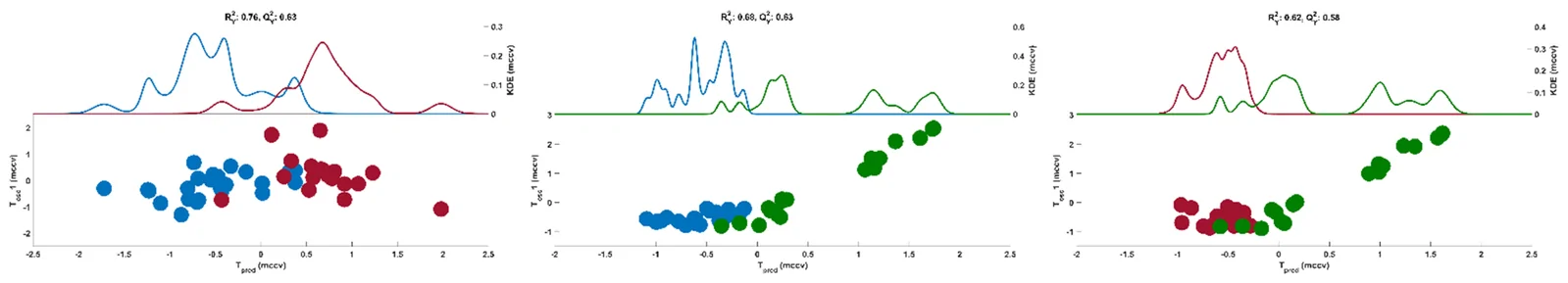
MCCV-OPLS-DA score plots derived from 1D ^1^H-NMR spectra of the metabotype of market-ripe oranges at different timepoints in a pairwise comparison (D9+D18, D30, and D35 in blue, red, and green, respectively). Each model comprises a Kernel Density Estimate (KDE) from the predicted scores (Tpred) for both groups. Dots represent the metabotype of each analysed orange. The fit and predictability of each model were obtained and expressed as R^2^Y (explained variance) and Q^2^Y (capability of prediction) values using two components in a 1 + 1 framework (i.e., one principal component and one orthogonal component). Comparisons made: D9+D18 (n = 25) vs. D30 (n = 18; (**left**)), D9+D18 (n = 25) vs. D35 (n = 19; (**middle**)), and D30 (n = 18) vs. D35 (n = 19; (**right**)).

Most MCCV-OPLS-DA models were statistically significant, exhibited adequate model performance, and showed no clear evidence of overfitting. Clear clustering separation across timepoints was easily visualised through Kernel Density Estimate (KDE, Figure 2 and Figure S1). The corresponding pairwise comparisons between timepoints resulted in good models, with Q^2^Y > 0.5 and with Δ < 0.3 (i.e., R^2^Y − Q^2^Y; Tables S2 and S3) [34,35].

However, comparing the metabotype of oranges with reduced turgor to those with firm pericarp tissue within D35 resulted in a non-significant model (R^2^Y = 0.31, Q^2^Y = 0.03), indicating that, despite their morphological differences, their metabotypes are similar (Figure S4). Furthermore, the pairwise model for sensitivity analysis, in which D18 was assessed separately by comparing D9 and D18, resulted in a poor model (R^2^Y = 0.77, Q^2^Y = 0.3 and D = 0.47). Based on the MCCV-OPLS-DA model and its corresponding KDE and predicted scores, this suggested that the orange metabotypes exhibited similar profiles at both timepoints. (Figure S1A). These two timepoints were therefore combined into one group, denoted D9+D18, and then compared with D30 and D35 using separate pairwise comparison models. For exact R^2^Y and Q^2^Y values for all pairwise models, please refer to Supplementary Tables S1 and S2.

**Figure 3:**
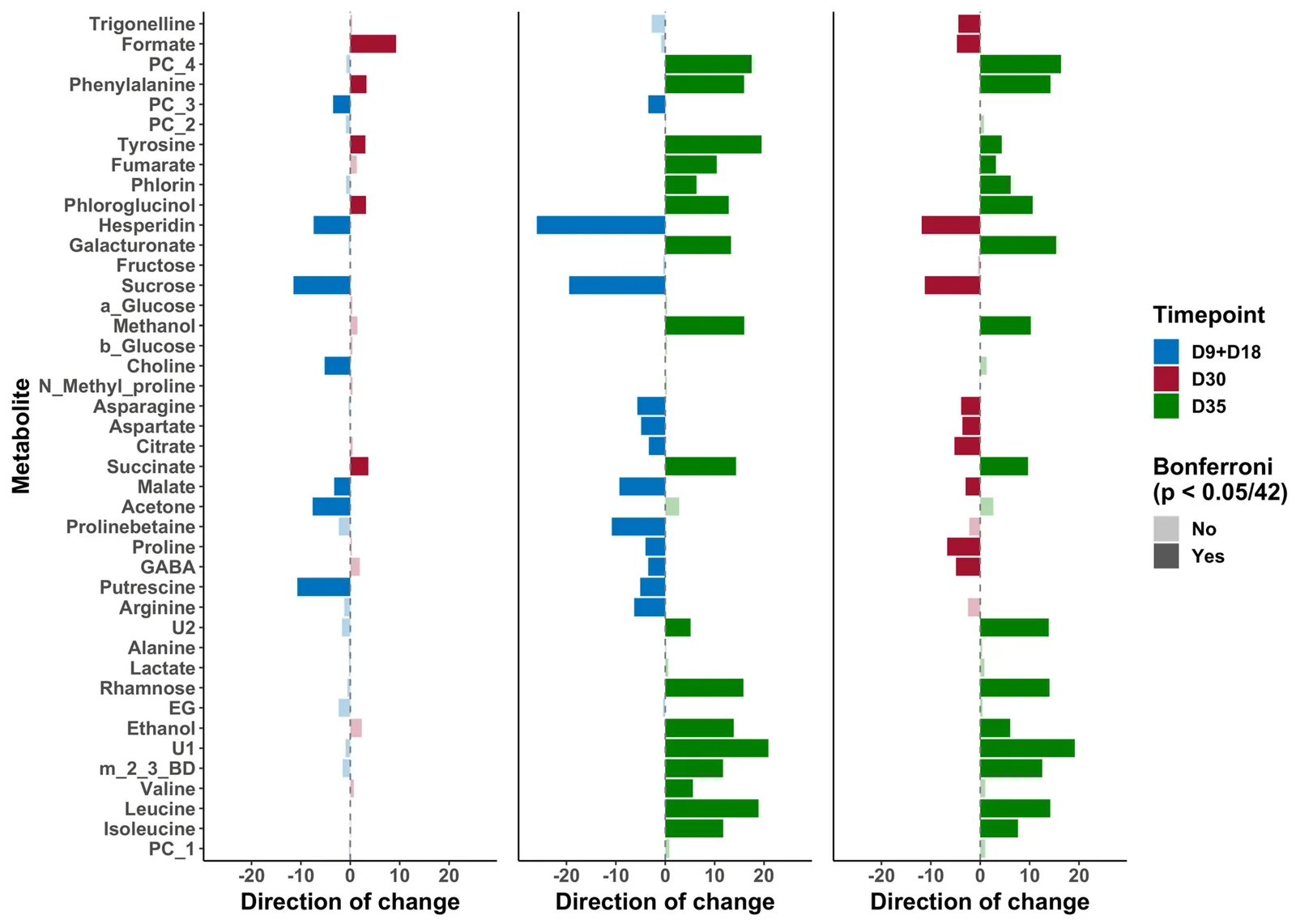
MCCV-OPLS-DA loading plots derived from the 1D ^1^H-NMR spectra of the metabotype of market-ripe oranges at different timepoints in a pairwise comparison (D9+D18, D30, and D35 in blue, red, and green, respectively). The X-axis depicts the direction of change derived from the variable significance detected per variable for each pairwise model. In blue are shown metabolites that are significantly higher at D9+D18 (n = 25); in red, metabolites that are significantly higher in D30 (n = 18), and in green, metabolites that are significantly higher at D35 (n = 19). Comparisons made: D9+D18 vs. D30 (**left**), D9+D18 vs. D35 (**middle**), and D30 vs. D35 (**right**). Significant changes were identified using Bonferroni correction with a threshold of *p* < 0.05/42. Key: GABA, 4-aminobutyrate; EG, ethyl glucoside; m-2,3-BD, *meso*-2,3-butanediol; PCs, phenolic compounds; Ui, unknown metabolites.

Metabolic changes were consistent across both analyses, merging D9 and D18, as well as the sensitivity analysis in which D18 was assessed separately. For instance, putrescine, acetone, malate, choline, sucrose, hesperidin and phenolic compound 3 were all increased at both D9+D18 and D9 when compared with D30. Succinate, tyrosine, phenylalanine and formate were increased at D30 when compared with D9+D18. However, ethanol, *N*-methyl-proline and fumarate were increased at D30 compared with D9; and phloroglucinol was the only metabolite increased at D30 compared with D9+D18 (Figure 3 and Figure S2, Tables S2 and S3).

At both D9+D18 and D9, arginine, putrescine, prolinebetaine, malate, citrate, aspartate, asparagine, sucrose, hesperidin and phenolic compound 3 were found to be increased against D35. GABA and proline were increased at D9+D18, while trigonelline was only increased at D9, compared with D35. Accordingly, isoleucine, leucine, valine, *meso*-2,3-butanediol, U1, ethanol, rhamnose, U2, succinate, methanol, galacturonate, phloroglucinol, phlorin, fumarate, tyrosine, phenylalanine and phenolic compound 4 were all increased at D35 in comparison with D9+D18 and D9. *N*-methyl-proline was the only compound to be increased at D35 compared with D9 (Figure 3 and Figure S2, Tables S2 and S3).

GABA, proline, malate, citrate, aspartate, asparagine, sucrose, hesperidin, formate, and trigonelline increased at D30 against D35. By contrast, isoleucine, leucine, *meso*-2,3-butanediol, U1, ethanol, rhamnose, U2, succinate, methanol, galacturonate, phloroglucinol, phlorin, fumarate, tyrosine, phenylalanine, and phenolic compound 4 increased at D35 against D30 (Figure 3 and Table S2).

Regarding the sensitivity analyses, putrescine and trigonelline were increased at D9 compared with D18. Conversely, leucine, valine, ethanol, *N*-methyl-proline, tyrosine and phenylalanine increased at the later timepoint. Choline and sucrose levels were higher at D18 than at D30, whereas formate showed an inverse trend. Lastly, arginine, GABA, proline, prolinebetaine, malate, asparagine, sucrose and hesperidin increased at D18 when compared with D35. However, isoleucine, leucine, *meso*-2,3-butanediol, U1, ethanol, rhamnose, succinate, methanol, galacturonate, phloroglucinol, phlorin, fumarate, tyrosine, phenylalanine and phenolic compound 4 increased at D35 (Figure S3, Table S3).

To better observe the patterns of significant metabolites across postharvest ageing stages, cross-sectional metabolite trends were plotted for both the primary and sensitivity analyses (Figures S7 and S8). A sustained decrease was observed across time for putrescine, sucrose, malate, prolinebetaine, proline, arginine, asparagine, hesperidin, and phenolic compound 3. On the other hand, a sustained increase was observed for phenolic compound 4, phloroglucinol, rhamnose, fumarate, galacturonate, phenylalanine, phlorin, U1, U2, tyrosine, isoleucine, valine, leucine, methanol, ethanol and succinate.

**Figure 4:**
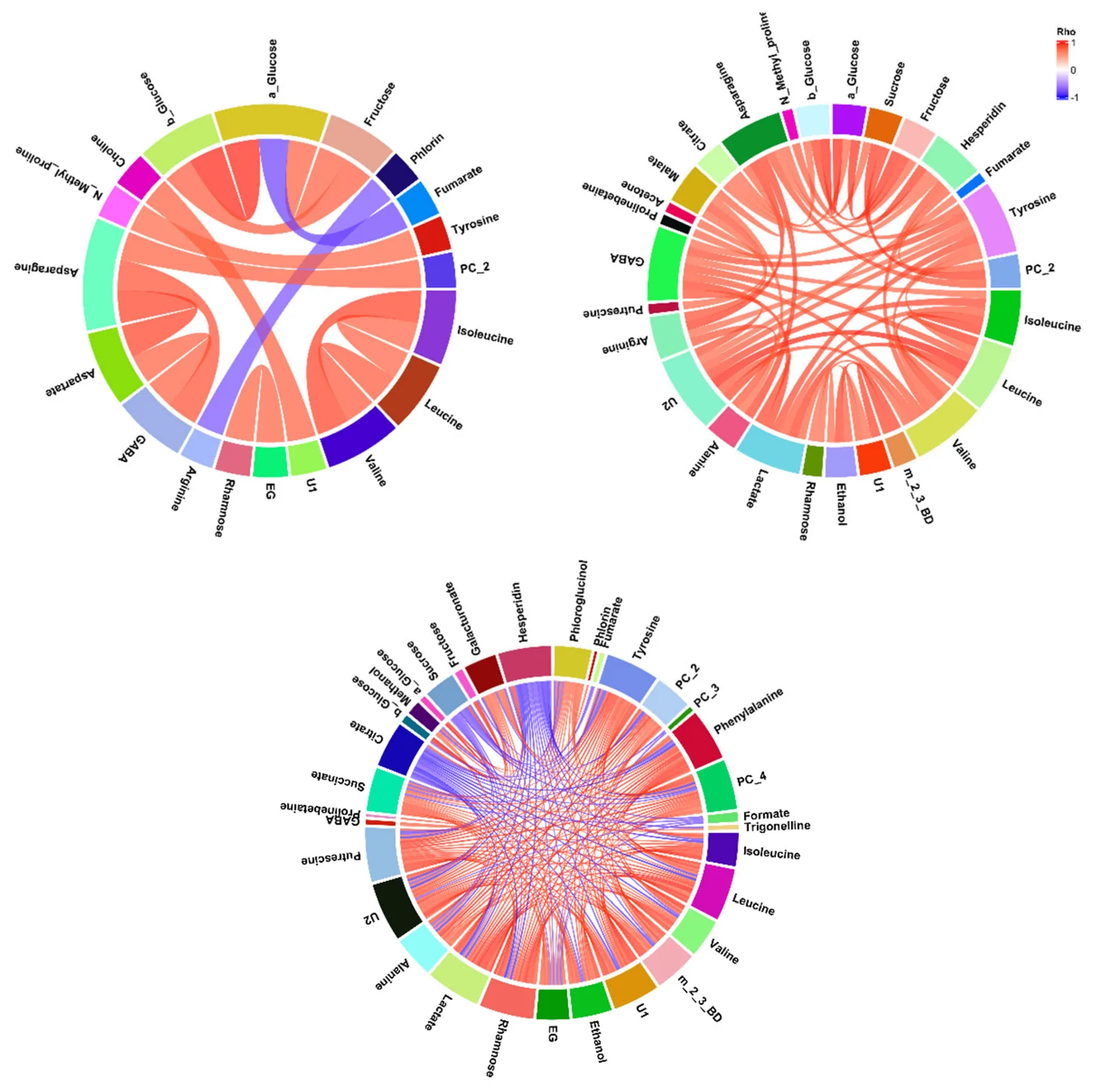
Correlations of metabolite NMR peak areas of market-ripe orange with |*rho*| ≥ 0.75 and *q*-value < 0.05 for D9+D18 (n = 25), D30 (n = 18) and D35 (n = 19; top left, top right, and bottom, respectively), showing 15, 50, and 210 significant correlations with the corresponding network densities of 1.74%, 5.81%, and 24.39%, respectively. Red denotes a positive correlation and blue a negative correlation. Key: GABA, 4-aminobutyrate; EG, ethyl glucoside; m-2,3-BD, *meso*-2,3-butanediol; PCs, phenolic compounds; Ui, unknown metabolites.

**Figure 5:**
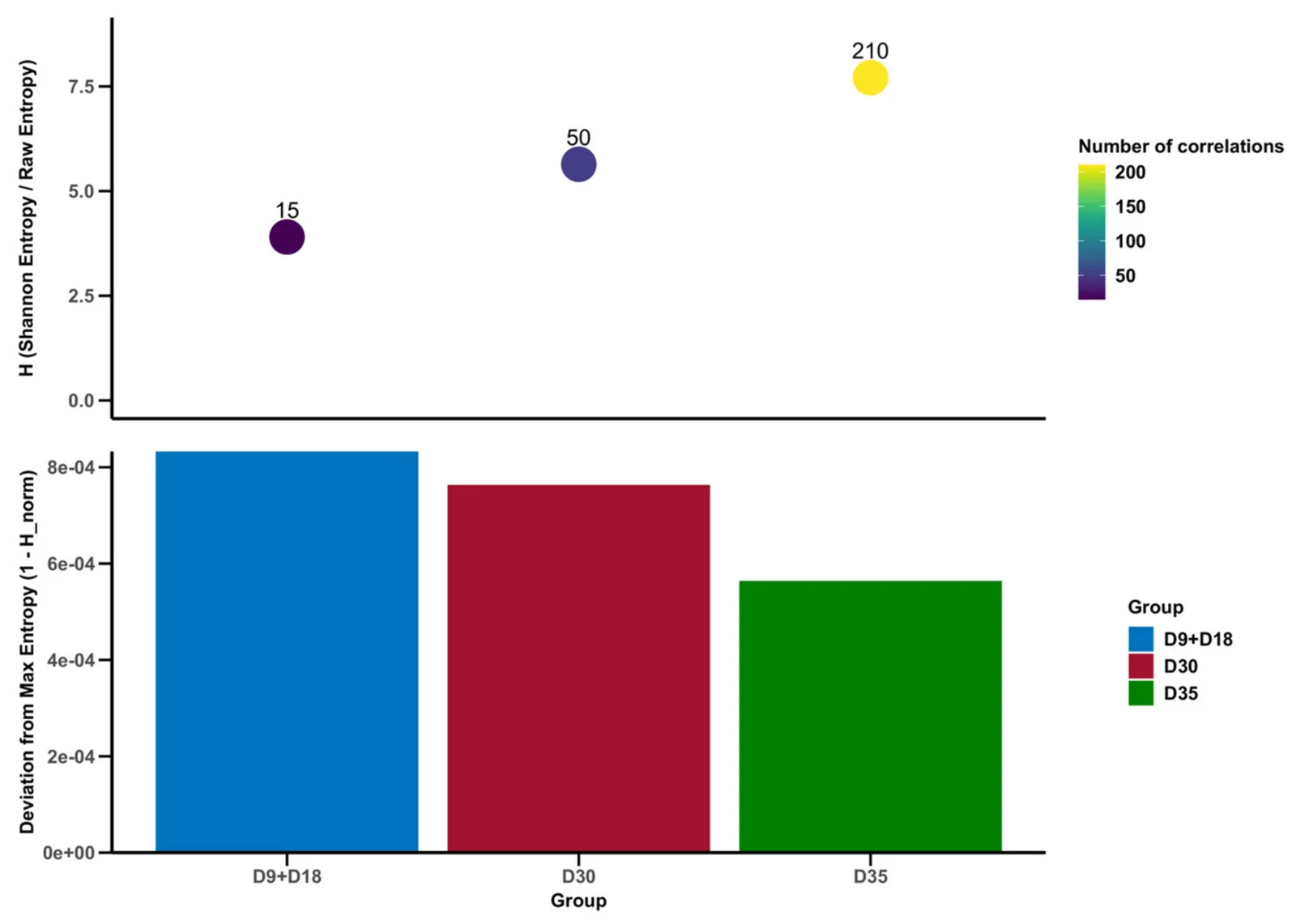
Entropy analysis. Shannon entropy (*H*, also known as raw entropy) coloured by the number of correlations per timepoint (top). Normalised entropy (*H_norm_*) is visualised as normalised deviation (1 − *H_norm_*) comparing D9+D18 (n = 25), D30 (n = 18), and D35 (n = 19; blue, red, and green, respectively; bottom). Entropy was calculated from only statistically significant correlations.

Furthermore, to detect patterns of orange metabolites per timepoint, correlation analysis was performed. Concomitantly, to evaluate whether the number of correlations was associated with metabolic complexity at each timepoint, entropy was calculated from the corresponding correlation matrices. The correlation structure differed in the number of metabolites and in the number of significant metabolite–metabolite correlations across timepoints (Figure 4 and Figure S5). For instance, 19, 27 and 33 metabolites characterised the correlation pattern at D9+D18, D30 and D35, respectively. The number of correlations also increased over time (e.g., 15, 50 and 210, in the same order). The correlation structure at D30 was defined by positive correlations only (Figure 5). For the sensitivity analysis of D9 and D18, 29 and 2 metabolites defined the correlation structure with 55 and 1 correlation, respectively (Figure S6). In this context, despite the relatively small sample size, all significant correlations were supported by bootstrap analysis, with 95% confidence intervals that did not cross zero, indicating that the direction and statistical evidence of these correlations were robust to resampling (Supplementary Tables S4–S9). Accordingly, raw Shannon entropy (*H*), which is related to absolute disorder, increases with the number of correlations. The normalised deviation (1 − *H_norm_*), which highlights subtle differences between groups and is related to relative disorder, has the opposite behaviour (Figure 5 and Figure S6). Entropy values could be calculated for all timepoints based on significant correlations, with D18 being the only exception, where a single significant association was identified and therefore did not provide a sufficient correlation structure for meaningful entropy calculation.

## 4. Discussion

### 4.1. The Rationale for Merging Days 9 and 18

The combination of these timepoints was supported by the main objective of this study, which focuses on the later stages of postharvest ageing and the quality deterioration experienced by consumers, rather than on mechanistic changes occurring at the earliest stages of storage. D18 was initially included to provide additional molecular information during the early postharvest period. However, the molecular profiles observed at this timepoint did not show substantial differences from those observed on D9. The experimental design was therefore developed with the final consumer in mind, focusing on quality changes relevant to fruit available on the market. As market-sourced oranges exhibit inherent heterogeneity, the samples were first screened to remove fruits with hidden defects that could have introduced bias into the NMR analysis. Following this quality-control step, and to maintain the most homogeneous sample size and group composition possible, D9 and D18 were combined. This decision was based on the comparable initial ripening/quality status observed in the two groups rather than on the OPLS-DA model alone. This grouping allowed us to focus the analysis on the progressive changes that occur during postharvest ageing and on the practical shelf life of the fruit under conditions relevant to consumers.

### 4.2. C. sinensis Metabotype over Time

The orange (*C. sinensis*) juice metabotype (here just “metabotype”) is characterised by carbohydrates (glucose, sucrose and fructose), essential amino acids (isoleucine, leucine, valine and phenylalanine), conditionally essential amino acids (arginine, proline and tyrosine), non-essential amino acids (alanine, aspartate and asparagine), alcohols (ethanol and methanol), polyphenols (hesperidin and unidentified phenolic compounds), and other metabolites that may have direct nutritional value (GABA, choline, trigonelline and prolinebetaine) [3,4,5,36] or indirect nutritional value as implicated in energy metabolism as intermediates (lactate, malate, succinate, fumarate and citrate), and metabolites with limited or no nutritional value (acetone and formate). These metabolites were detected from D9, representing the early-ageing metabotype.

Our results, obtained by modelling the NMR data using MCCV-OPLS-DA, indicate that the abundance of some of these metabolites changed or that degradation products were produced over time during shelf storage at room temperature, compared with the baseline orange metabotype. For instance, the levels of formate, phenylalanine, tyrosine, phloroglucinol, and succinate increased, while the levels of hesperidin, sucrose, putrescine, acetone and malate decreased at D30. In line with this, branched-chain amino acids (BCAAs, leucine, isoleucine, valine), ethanol, methanol, fumarate, phlorin, rhamnose, *meso*-2,3-butanediol, galacturonate, phenolic compound 4, and the aliphatic compounds U1 and U2 increased, whereas asparagine, aspartate, citrate, prolinebetaine, proline, GABA and arginine increased at D35. Meanwhile, fructose, glucose, alanine, lactate, ethyl glucoside, and phenolic compounds 1 and 2 remained unchanged over time. Taken together, these findings suggest metabotype patterns of ageing and advanced ageing at days 30 and 35, respectively. An overview of the changes derived from postharvest ageing reflected in the metabotype is shown by plotting cross-sectional metabolite trends (Figures S7 and S8).

These patterns were consistent with the correlation structure of the orange metabotypes at both time points. For instance, the highest number of correlations was observed at D35. Hesperidin and citrate were negatively correlated with metabolites related to advanced ageing that were observed to be increased at either D30 or D35 compared with D9/D9+D18. These metabolites include rhamnose, phenolic compound 4, alanine, phenylalanine, tyrosine, BCAAs, lactate, succinate, phloroglucinol, putrescine, ethanol, ethyl glucoside, *meso*-2,3-butanediol, U1 and U2. Additionally, hesperidin showed negative correlations with galacturonate and phenolic compound 2, as well as positive correlations with citrate and sucrose. Concomitantly, the latter had negative correlations with rhamnose, phenolic compounds 2 and 4, and the amino acids alanine, phenylalanine and tyrosine. It also had negative correlations with lactate, phloroglucinol, putrescine, methanol, galacturonate and U2. Moreover, metabolites that are inversely correlated with hesperidin, citrate, and sucrose tend to be positively correlated with each other. This suggests that these metabolites shared similar patterns of variation. Most of these metabolites that produced inverse correlations with hesperidin, citrate and sucrose are considered degradation byproducts.

For instance, hesperidin can degrade to produce rutinose, which undergoes complete hydrolysis to produce rhamnose and glucose. Meanwhile, phenolic compounds 2 and 4 could be degradation byproducts of hesperetin. Ethanol and methanol may be produced through the fermentation of sugars, such as sucrose, rutinose, rhamnose and glucose. Galacturonate is another degradation byproduct from carbohydrates. Phenylalanine, tyrosine, and phenolic compounds 2 and 4 could derive from the degradation of hesperidin. Furthermore, BCAAs, phenylalanine, tyrosine and alanine may be produced through protein degradation by enzymes or through microbial fermentation. The presence of phlorin and its possible degradation product, phloroglucinol, may be related to the changes in the pericarp tissue, which facilitate their transfer into the juice. This occurs despite careful handling to avoid overextraction when collecting the sample. Regarding the correlation analysis at D30, this timepoint showed the second-highest number of correlations. Its structure consists entirely of positive correlations, which makes it particularly distinct compared to the correlation structures at the other timepoints.

The resulting metabolite–metabolite correlations were used to identify patterns of covariation among metabolites and were not interpreted, by themselves, as evidence of direct structural or biosynthetic relationships.

Although the correlations do not directly reflect structural relationships among the metabolites, the correlation patterns were used to obtain entropy values for each timepoint, revealing a progressive increase in metabolic complexity across the postharvest ageing, reaching its highest at D35.

### 4.3. Nutritional and Commercial Implications

One objective for prolonging the post-harvest storage of citrus fruits is to delay deterioration, thereby ensuring postharvest quality, consumer acceptability, commercial value, and prolonging shelf life. In this sense, and to the best of our knowledge, this is the first study to characterise metabolite abundance and metabolic patterns during postharvest ageing of market-ripe *C. sinensis* at room temperature. NMR-based metabolic phenotyping made it possible to distinguish the early-ageing, ageing and advanced-ageing metabotypes. Concomitantly, the progressive metabotype complexity corresponded to the changes in the fruit’s morphological characteristics over time. For instance, on day 9, the fruits were generally yellow to green in colour, had turgor, were uniform in size, were firm to the touch, and produced yellow juice. On day 30, all oranges had turned yellow and the juice was darker than in previous timepoints. Similar morphological changes have been reported in association with cellular deterioration in fruit [37,38]. Therefore, this timepoint defined an ageing metabotype. By day 35, the morphological characteristics of the fruit were already very different compared with those from the other timepoints. Two subgroups were identified: one with reduced turgor and the other with firm pericarp tissue. However, the former showed a subtle unpleasant smell, suggesting possible microbial fermentation, which was not observed in the latter. Despite these changes, the resulting juice exhibited pronounced browning in both. This colour alteration is consistent with advanced fruit deterioration and may arise from multiple processes associated with ageing. At advanced stages of postharvest deterioration, microbial infection of *C. sinensis* by *Penicillium* spp. has been associated with substantial changes in primary metabolism and volatile profiles, including increased ethanol accumulation and a concomitant decline in fruit quality [39]. Therefore, the accumulation of ethanol observed in the advanced-ageing metabotype may reflect enhanced fermentative metabolism and/or pathogen-associated deterioration, whereas changes in methanol and formate should be interpreted cautiously because they may originate from additional metabolic and cell-wall degradation processes. Accordingly, this timepoint was characterised by a metabotype with advanced ageing and the highest entropy values, consistent with greater metabolic complexity.

The behaviour observed in the metabotype after postharvest using NMR was similar to what was reported using other techniques, indicating a natural mechanism in citrus fruits after harvest [40], where non-climacteric fruits exhibit minimal changes due to ripening, but continue to undergo ageing. This results in a deterioration of quality during storage. For instance, citrate catabolism is associated with an increased presence of alcohols and aldehydes, as well as other secondary metabolites, which are linked to a decrease in the juice flavour in hot climates [41]. However, aliphatic alcohols, such as ethanol and methanol, have not yet been observed in the analysis. Furthermore, citrate is a product of the Krebs cycle that accumulates in vacuoles during fruit development and decreases during ripening and maturation [42,43]. Citrate accounts for over 90% of the total acid content, whereas other acids, such as malate, are present in low concentrations.

Although the mechanisms underlying changes in citrate abundance and its degradation during pre- and postharvest are unknown, citrate plays a crucial role in the quality of many fruit species, especially citrus fruits [43].

The presence of aspartate, asparagine, serine, glutamine and proline has also been reported in orange juice from ripe fruit [16,44]. Tyrosine and phenylalanine are found in lower proportions but undergo increased biosynthesis under biotic and abiotic stress conditions. This is consistent with the biosynthesis of flavonoids, such as hesperidin, as well as other phenolic compounds. These secondary metabolites have antioxidant properties and are derived from hydroxycinnamic acids, whose biosynthesis involves tyrosine and phenylalanine as precursors [45]. Alternatively, the presence of these amino acids could also indicate the degradation of polyphenols, as observed on days 30 and 35. Tyrosine and phenylalanine are considered antioxidants *per se* and can prevent cell damage caused by reactive oxygen species, which are generated by the fruit as a defence mechanism and as signalling molecules that induce cell death [45]. Therefore, their increasing abundance may be associated with the cellular stress experienced by the fruit during postharvest ageing. Although microbial communities were not assessed in the present study, the potential involvement of *Penicillium* spp. may be considered based on previous reports. In particular, *Penicillium digitatum* has been shown to suppress the enzyme phenylalanine ammonium lyase activity in citrus fruit, potentially affecting phenylpropanoid biosynthesis and related host-defence responses [46]. This mechanism may contribute to changes in phenylalanine levels; however, it does not directly explain the observed tyrosine increase. Both metabolites may instead reflect broader changes in aromatic amino acid metabolism associated with postharvest stress. These interpretations remain speculative and require direct experimental validation. Furthermore, BCAAs also act as defence metabolites against pathogens [47]. These amino acids were also increased in the ageing and advanced-ageing metabotypes.

During citrus fruit development and ripening, soluble sugars progressively accumulate, with sucrose, glucose, and fructose representing the predominant carbohydrates [43,48]. Sucrose homeostasis is controlled by coordinated transport, synthesis, and cleavage mechanisms, including sucrose transporters, sucrose synthases, and vacuolar and cell-wall invertases [47]. During the advanced postharvest stage, increased carbohydrate catabolism may promote glycolytic and fermentative metabolism, resulting in the accumulation of ethanol, particularly under tissue stress or pathogen-associated decay [39]. Methanol accumulation, however, should be considered separately from ethanol fermentation. In citrus-derived products, methanol can arise from the de-esterification of methylated pectins through pectin methylesterase activity and may increase during tissue degradation or microbial processing [49]. Consistent with progressive cell-wall disassembly, pectin solubilisation, de-esterification, and depolymerisation during citrus storage can also increase galacturonic-acid-related metabolites [50]. Changes in the volatile fraction, including alcohols, aldehydes, esters, and ketones, accompany citrus maturation, storage, and pathogen-associated deterioration and may contribute to changes in aroma and off-flavour development [51].

Several studies indicate that the catabolism of organic acids and amino acids, as well as carbohydrate metabolism, are interconnected. A report suggests that the decrease in citrate is due to its utilisation in three metabolic pathways: sugar production, amino acid synthesis and acetyl-CoA generation [18,52]. Consistent with the progressive shift from organic acid accumulation to sugar accumulation during fruit development, recent studies have shown that sucrose, glucose, and fructose progressively accumulate in citrus fruit, with sucrose becoming a major component of the soluble sugar pool during ripening [43]. In the present study, citrate showed negative correlations with several amino acids and organic acids, whereas a positive correlation was observed with sucrose. This relationship may reflect the coordinated remodelling of organic acid and carbohydrate metabolism during citrus fruit development rather than a direct metabolic conversion between citrate and sucrose. Previous proteomic and metabolomic analyses have also shown that citrate decreases during the final stages of citrus fruit development, accompanied by substantial changes in tricarboxylic acid cycle intermediates and amino acid metabolism [53]. These observations are consistent with the metabolic trends identified in the present study.

The metabolomic composition of orange juice is highly complex, and its complete characterisation remains analytically challenging. In the present study, NMR-based metabolic phenotyping enabled the detection of 36 identified and six currently unidentified metabolites, of which two were tentatively classified as aliphatic compounds and four as phenolic compounds based on their chemical shifts and signal multiplicities. Nevertheless, additional metabolites may be present at concentrations below the detection limit of NMR spectroscopy or may remain unresolved because of spectral overlap, thereby limiting complete coverage of the orange juice metabotype.

Despite these analytical limitations, the metabolites identified in the present study provide a basis for developing targeted approaches for assessing orange juice quality and authenticity. Recent studies have demonstrated that targeted ^1^H-NMR spectroscopy, particularly when combined with complementary chromatographic techniques, can characterise citrus juices [54]. Accordingly, metabolites identified in the present study, including phlorin, galacturonate, succinate, rhamnose, and proline betaine, could be further evaluated as components of targeted biomarker panels for orange juice quality and authentication.

In addition, prolinebetaine is particularly relevant because it is abundant in citrus products and has been extensively validated as a biomarker of citrus consumption, including orange juice intake [55]. However, because prolinebetaine is not exclusive to *C. sinensis*, its potential application to orange juice authentication should preferably be considered as part of a multimetabolite metabolic panel rather than as a stand-alone, species-specific biomarker. Such targeted approaches are consistent with recent developments in citrus juice authentication, in which combinations of metabolomic markers and complementary analytical platforms are increasingly used to improve species discrimination and adulteration detection [56,57].

A potential limitation of the study is that acquisition order was not randomised across postharvest ageing timepoints, and therefore a potential acquisition-order effect cannot be completely excluded. Nevertheless, the acquisition sequence (D9, D18, D35, D30) did not follow the chronological order of postharvest ageing, reducing the extent to which acquisition time and ageing time were systematically aligned. In addition, all spectra underwent identical preprocessing and PQN normalisation, and the robustness of the OPLS-DA models was evaluated using repeated random MCCV. Together, these measures provide some protection against potential acquisition-related effects, although they cannot fully rule out residual confounding between acquisition order and postharvest ageing time.

## 5. Conclusions

This study provides a comprehensive characterisation of metabotype patterns associated with market-ripe *C. sinensis* postharvest ageing. Oranges intended for human consumption and available through a retail outlet were selected to simulate the storage process commonly occurring in households at room temperature. The observed changes in the metabolic profile demonstrate that the ageing process is associated with coordinated remodelling of primary and secondary metabolism, reflecting the progressive changes occurring in pathways related to energy metabolism, carbohydrate turnover, the dynamics of amino acids, organic acids and polyphenols. It also reflects the accumulation or depletion of metabolites associated with fruit postharvest ageing. This research demonstrates that comparative metabolic phenotyping across postharvest stages could identify key ageing-associated metabolites of market-ripe *C. sinensis*, including phlorin, galacturonate, succinate, rhamnose and prolinebetaine. In the future, this approach could be further developed into a compact and affordable device for rapid in situ monitoring of ageing-associated metabolites and/or combining metabolic phenotyping with measurements of physical, physicochemical and sensory quality to support the assessment of postharvest ageing status and fruit quality. Our findings also provide a metabolic basis for targeted strategies to monitor postharvest ageing and preserve the quality of perishable fruits and food products, with potential applications in the development and implementation of innovative preservation technologies.

## Data Availability

The data presented in this study are available on request from the corresponding authors.

## Supplementary Materials

The following supporting information can be downloaded at: https://www.mdpi.com/article/10.3390/metabo16100735/s1. Figure S1. MCCV-OPLS-DA score plots derived from 1D ^1^H-NMR spectra of the metabotype of market-ripe oranges at different timepoints in a pairwise comparison (D9, D18, D30 and D35 in blue, black, red, and green, respectively); Figure S2. MCCV-OPLS-DA loading plots derived from 1D ^1^H-NMR spectra of the metabotype of market-ripe oranges at different timepoints in a pairwise comparison (D9, D18, D30 and D35 in blue, black, red, and green, respectively) for sensitivity analysis; Figure S3. MCCV-OPLS-DA loading plots derived from 1D ^1^H-NMR spectra of the metabotype of market-ripe oranges at different timepoints in a pairwise comparison (D9, D18, D30 and D35 in blue, black, red, and green, respectively) for sensitivity analysis; Figure S4. MCCV-OPLS-DA score plot derived from 1D ^1^H-NMR spectra of the metabotype of market-ripe oranges within D35 (n = 19) comparing the juice produced from oranges that exhibited reduced turgor (n = 11; yellow) versus those with firm pericarp tissue (n = 8; orange); Figure S5. Correlations of market-ripe orange metabolite NMR peak areas with |rho| ≥ 0.75 and *q*-value < 0.05 for D9 (n = 17) regarding the sensitivity analysis, with 55 significant correlations and a network density of 6.39%; Figure S6. Entropy analysis for the sensitivity analysis; Figure S7. Cross-sectional metabolite trends (median ± IQR) for those metabolites whose change was significant at least in one pairwise MCCV-OPLS-DA model, including D9+D18 (n = 25), D30 (n = 18), and D35 (n = 19); Figure S8. Cross-sectional metabolite trends (median ± IQR) for those metabolites whose change was significant in at least one pairwise MCCV-OPLS-DA model, including D9 (n = 17), D18 (n = 8), D30 (n = 18), and D35 (n = 19) for the sensitivity analysis; Table S1. ^1^H and ^13^C NMR peak assignments for identified metabolites in orange (*C. sinensis*) juice; Table S2. Metabolites observed in orange (*C. sinensis*) whose changes in abundance were significantly associated with the differences observed across all timepoints; Table S3. Metabolites observed in orange (*C. sinensis*) whose changes in abundance were significantly associated with the differences observed across all timepoints for sensitivity analysis; Table S4. Correlation analyses for D9; Table S5. Correlation analyses for D18; Table S6. Correlation analyses for D30; Table S7. Correlation analyses for D35; Table S8. Correlation analyses for D9+D18; Table S9. Summary of network densities.

## Abbreviations

BCAAs	Branched-chain amino acids
DoC	Direction of change
EG	Ethyl glucoside
GABA	4-Aminobutyrate
KDE	Kernel density estimate
m-2,3-BD	meso-2,3-butanediol
MCCV	Monte-Carlo cross-validation
NMR	Nuclear magnetic resonance
OPLS-DA	Orthogonal projection to latent structures discriminant analysis
PCs	Phenolic compounds
