# Supplementary material for: Metabotype Dynamics of Market-Ripe Orange (Citrus sinensis) During Postharvest Ageing at Room Temperature
Source: Metabolites. 2026 Sep 30;16(10):735. doi: 10.3390/metabo16100735 (PMC13706830; doi:10.3390/metabo16100735)
Supplement: Supplementary file 1 [file metabolites-16-00735-s001.zip › Supplementary_Information_R2_Tracked.pdf]

Metabotype Dynamics of Market-Ripe Orange (*Citrus sinensis*) During Postharvest Ageing at Room Temperature

Mabel M. Montenegro-Sustaita [1], José Iván Serrano-Contreras [2,*], Gabriela Torres-Santiago [3] and Luis Gerardo Zepeda-Vallejo [1,*]

[1] Departamento de Química Orgánica, Escuela Nacional de Ciencias Biológicas, Instituto Politécnico Nacional, Prolongación de Carpio y Plan de Ayala s/n, Col. Santo Tomas, Delegación Miguel Hidalgo, Ciudad de Mexico 11340, Mexico

[2] Department of Metabolism, Digestion and Reproduction, Division of Digestive Diseases, Section of Nutrition, Faculty of Medicine, Imperial College London, London W12 0NN, UK

[3] Facultad de Ciencias Químicas, Circuito Universitario s/n, Campus Uach II, Chihuahua, Chih. 31125, Mexico

* Correspondence: j.serrano-contreras@imperial.ac.uk (J.I.S.-C.); lzepeda@ipn.mx (L.G.Z.-V.)

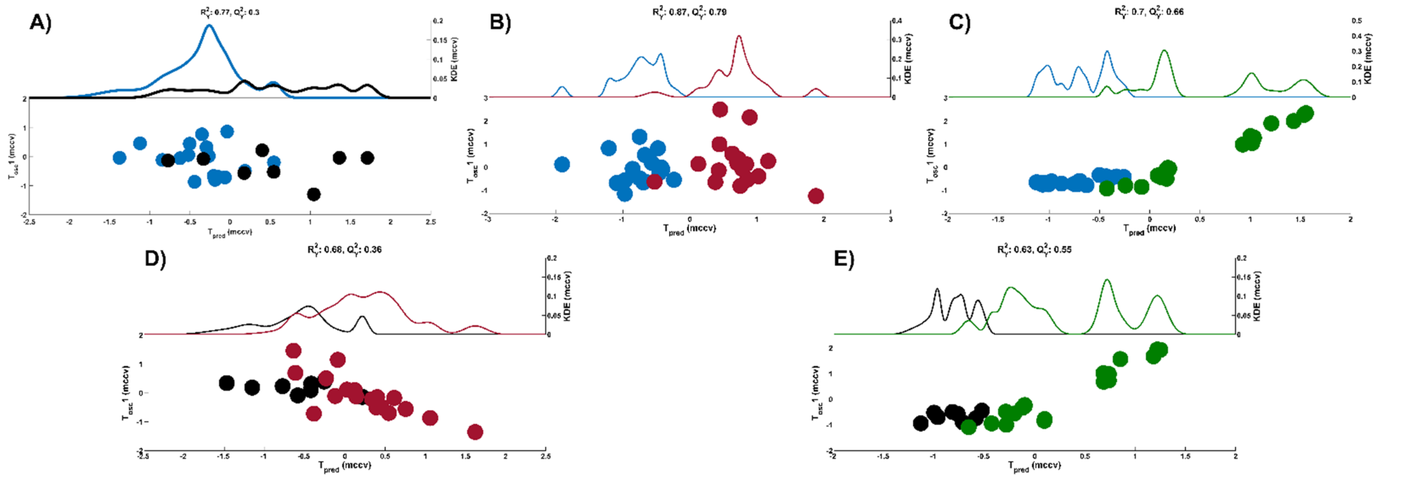

Figure S1. MCCV-OPLS-DA score plots derived from 1D ^{1}H-NMR spectra of the metabotype of market-ripe oranges at different timepoints in a pairwise comparison (D9, D18, D30 and D35 in blue, black, red, and green, respectively). Each model is comprised of a Kernel Density Estimate (KDE) from the predicted scores (Tpred) for both groups. Dots represent the metabotype of each orange analysed. The fit and predictability of each model were obtained and expressed as R^2Y (explained variance) and Q^2Y (capability of prediction) values using 2 components in a 1+1 framework (i.e., one principal component and one orthogonal component). Comparisons made: D9 (n = 17) vs D18 (n = 18; **A**), D9 (n = 17) vs D30 (n = 18, **B**), D9 (n = 17) vs D35 (n = 19; **C**), D18 (n = 8) vs D30 (n = 18, **D**), and D18 (n = 8) and D35 (n = 19; **E**).

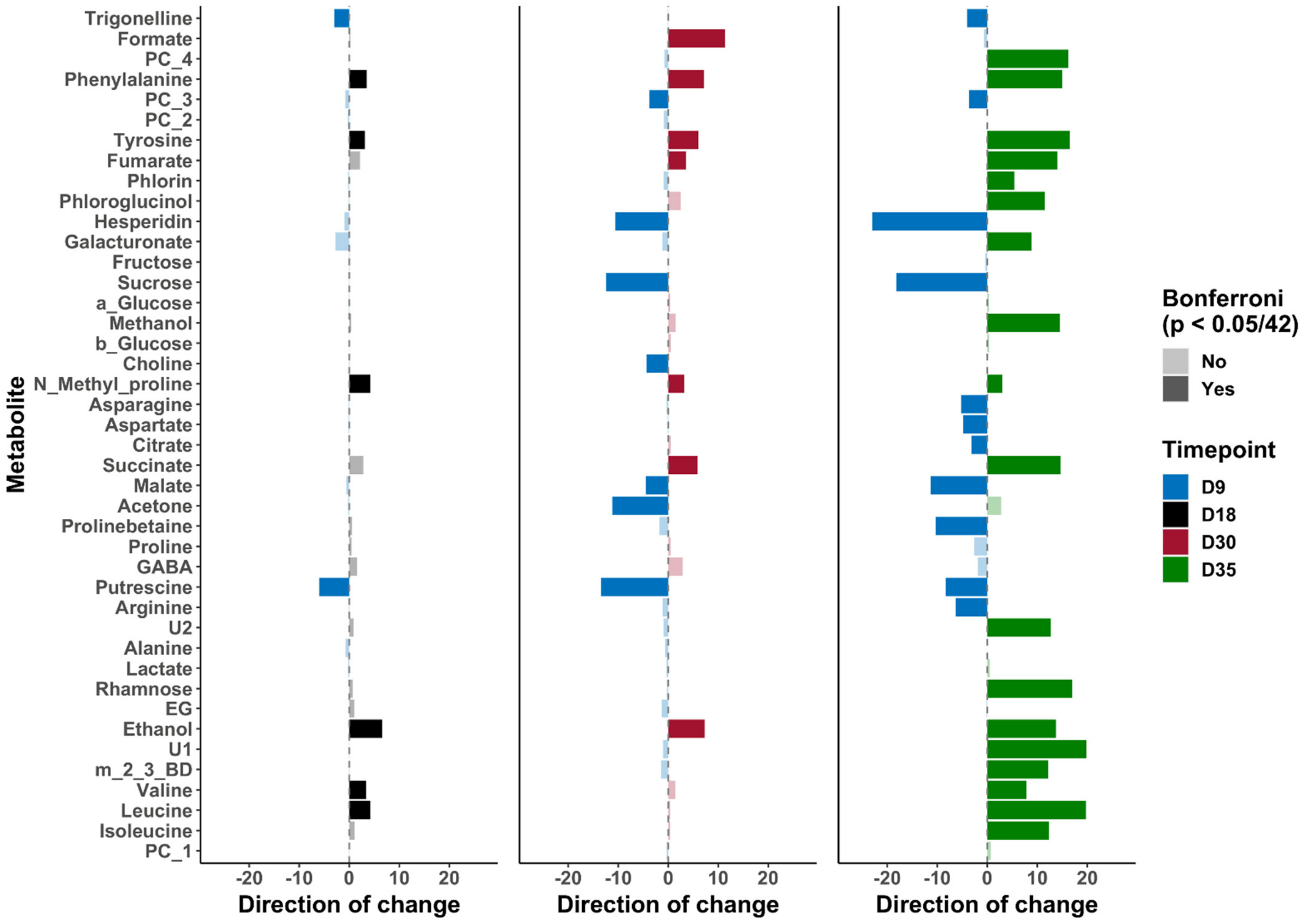

Figure S2. MCCV-OPLS-DA loading plots derived from 1D ^{1}H-NMR spectra of the metabotype of market-ripe oranges at different timepoints in a pairwise comparison (D9, D18, D30 and D35 in blue, black, red, and green, respectively) for sensitivity analysis. The X-axis depicts the direction of change derived from the variable significance detected per variable for each pairwise model. In blue, metabolites are shown that are significantly higher at D9 (n = 17), in black metabolites significantly higher in D18 (n = 8), in red metabolites significantly higher in D30 (n = 18), and in green metabolites significantly higher in D35 (n = 19). Comparisons made: D9 vs D18 (left), D9 vs D30 (middle), and D9 and D35 (right). Significant changes were identified using Bonferroni correction with a threshold of $p < 0.05/42$. Key: GABA, 4-aminobutyrate; EG, ethyl glucoside; m-2,3-BD, *meso*-2,3-butanediol; PCs, phenolic compounds; Ui, unknown metabolites.

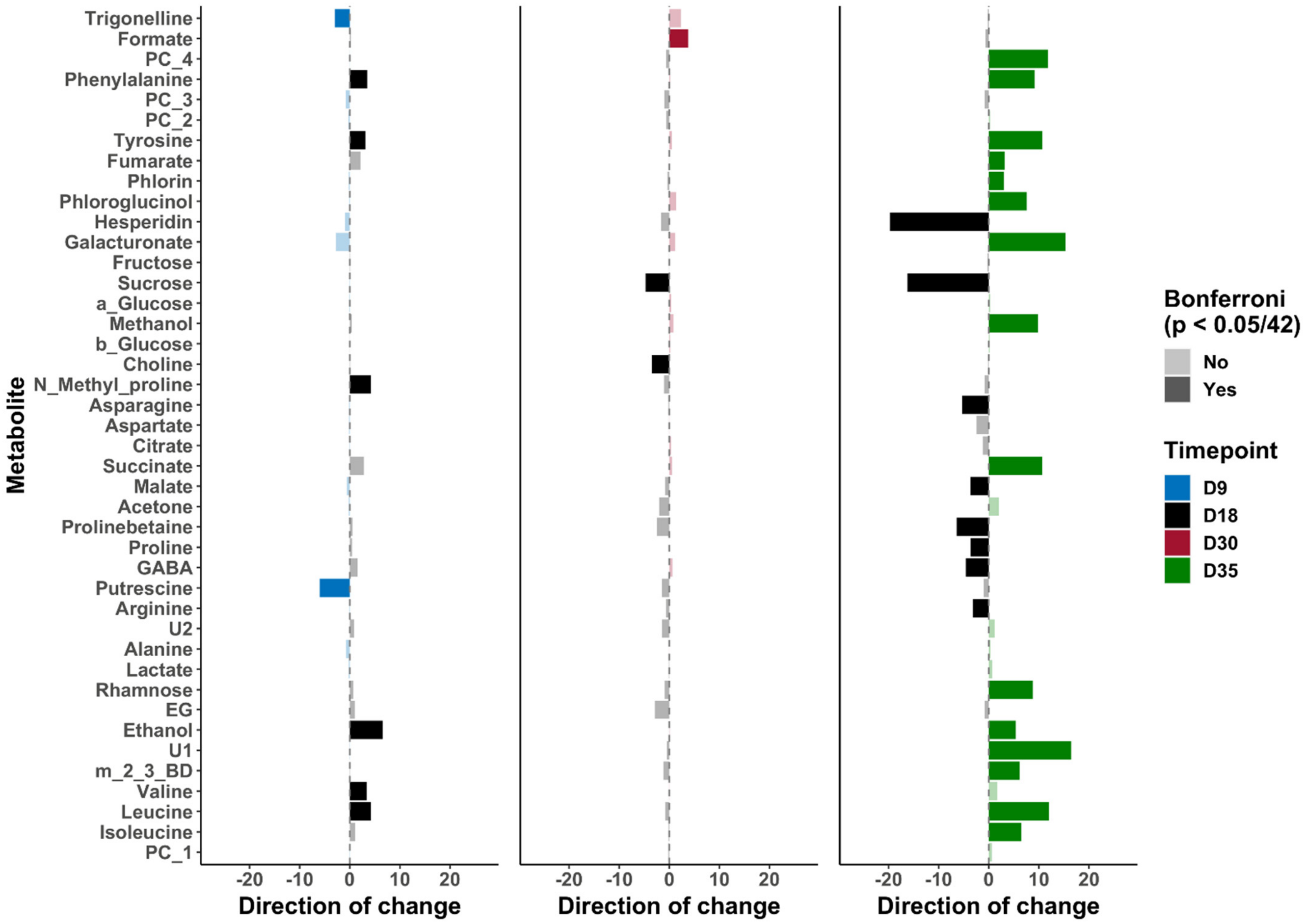

Figure S3. MCCV-OPLS-DA loading plots derived from 1D ^{1}H-NMR spectra of the metabotype of market-ripe oranges at different timepoints in a pairwise comparison (D9, D18, D30 and D35 in blue, black, red, and green, respectively) for sensitivity analysis. The X-axis depicts the direction of change derived from the variable significance detected per variable for each pairwise model. In blue, metabolites are shown that are significantly higher at D9 (n = 17), in black metabolites significantly higher in D18 (n = 8), in red metabolites significantly higher in D30 (n = 18), and in green metabolites significantly higher in D35 (n = 19). Comparisons made: D9 vs D18 (left), D18 vs D30 (middle), and D18 and D35 (right). Significant changes were identified using Bonferroni correction with a threshold of $p < 0.05/42$. Key: GABA, 4-aminobutyrate; EG, ethyl glucoside; m-2,3-BD, *meso*-2,3-butanediol; PCs, phenolic compounds; Ui, unknown metabolites.

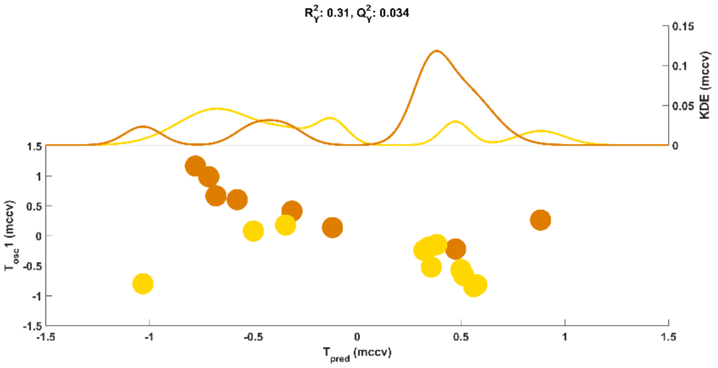

Figure S4. MCCV-OPLS-DA score plot derived from 1D ^{1}H-NMR spectra of the metabotype of market-ripe oranges within D35 (n = 19) comparing the juice produced from oranges that exhibited reduced turgor (n = 11; yellow) versus those with firm pericarp tissue (n = 8; orange). KDE, Kernel Density Estimate; Tpred predicted scores.

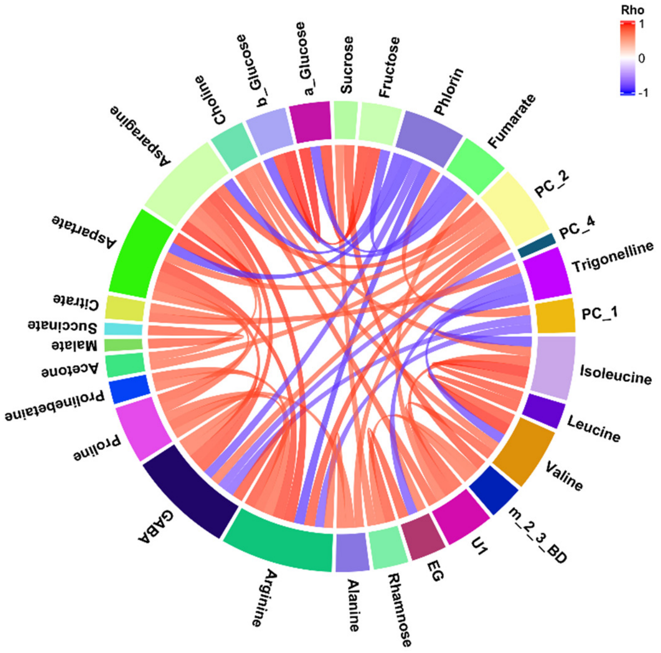

Figure S5. Correlations of market-ripe orange metabolite NMR peak areas with |*rho*| ≥ 0.75 and *q*-value < 0.05 for D9 (n = 17) regarding the sensitivity analysis, with 55 significant correlations with a network density of 6.39 %. Red denotes a positive correlation and blue a negative correlation. Key: GABA, 4-aminobutyrate; EG, ethyl glucoside; m-2,3-BD, *meso*-2,3-butanediol; PCs, phenolic compounds; Ui, unknown metabolites.

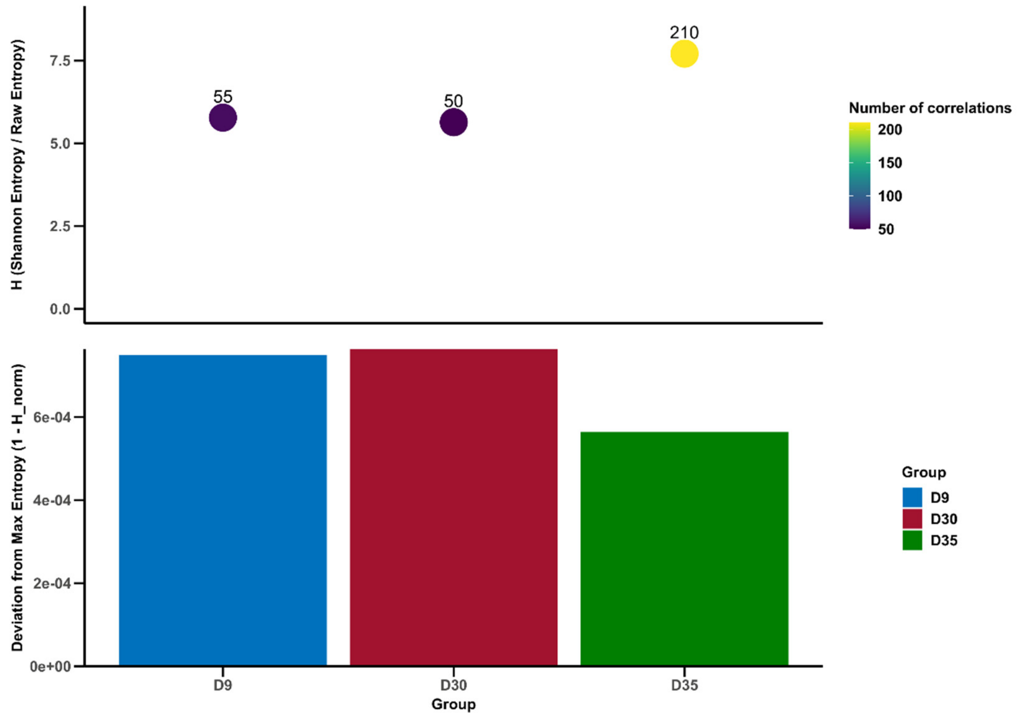

Figure S6. Entropy analysis for the sensitivity analysis. Shannon entropy (H, also known as raw entropy) coloured by the number of correlations per timepoint (top), along with normalised entropy (H_{norm}) visualised as normalised deviation ($1 - H_{norm}$) comparing D9 (n = 17), D30 (n = 18) and D35 (n = 19; blue, black, red, and green, respectively; bottom). Entropy was calculated from only statistically significant correlations.

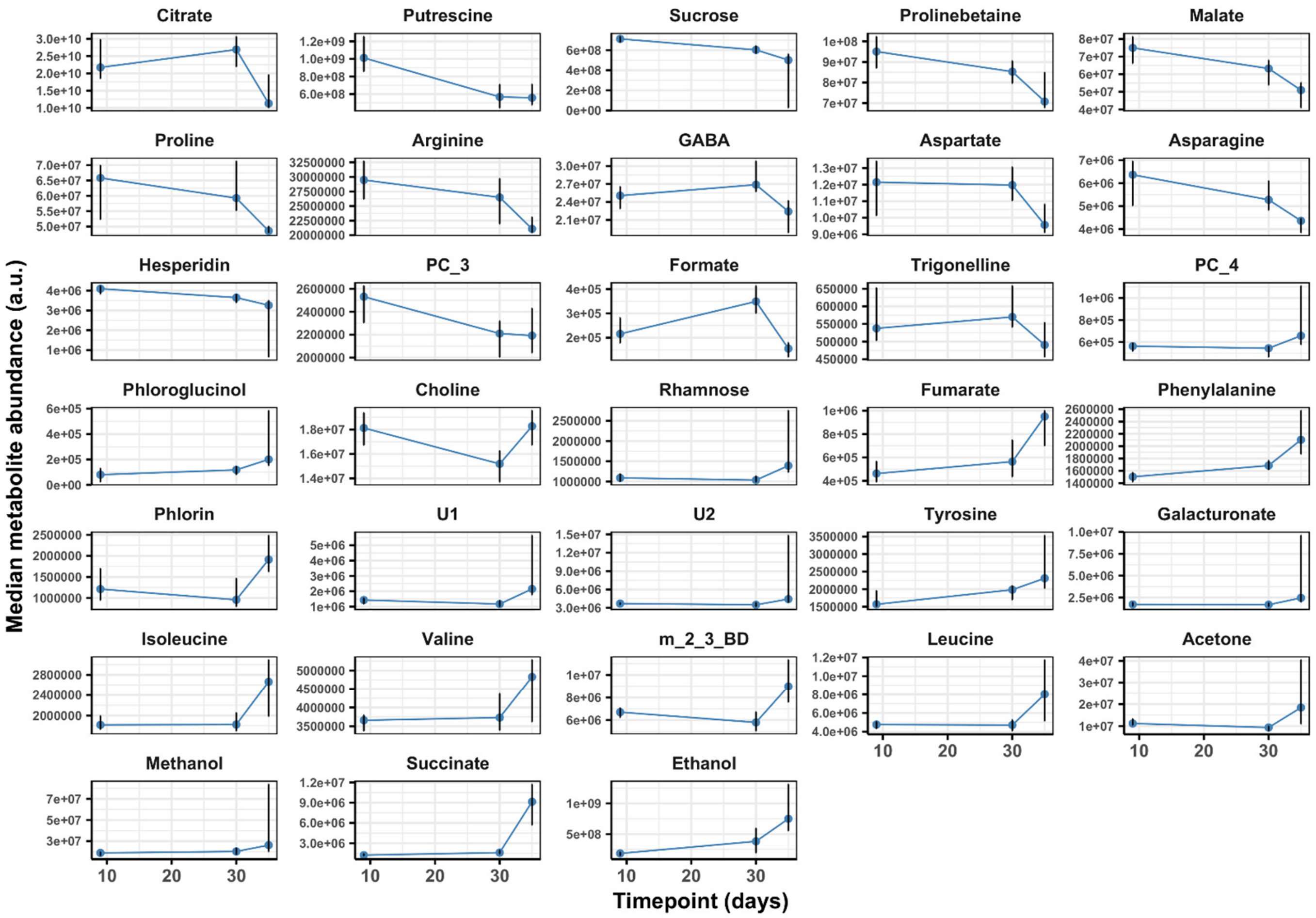

Figure S7. Cross-sectional metabolite trends (median ± IQR) for those metabolites whose change was significant at least in one pairwise MCCV-OPLS-DA model including D9+D18 (n = 25), D30 (n = 18), and D35 (n = 19).

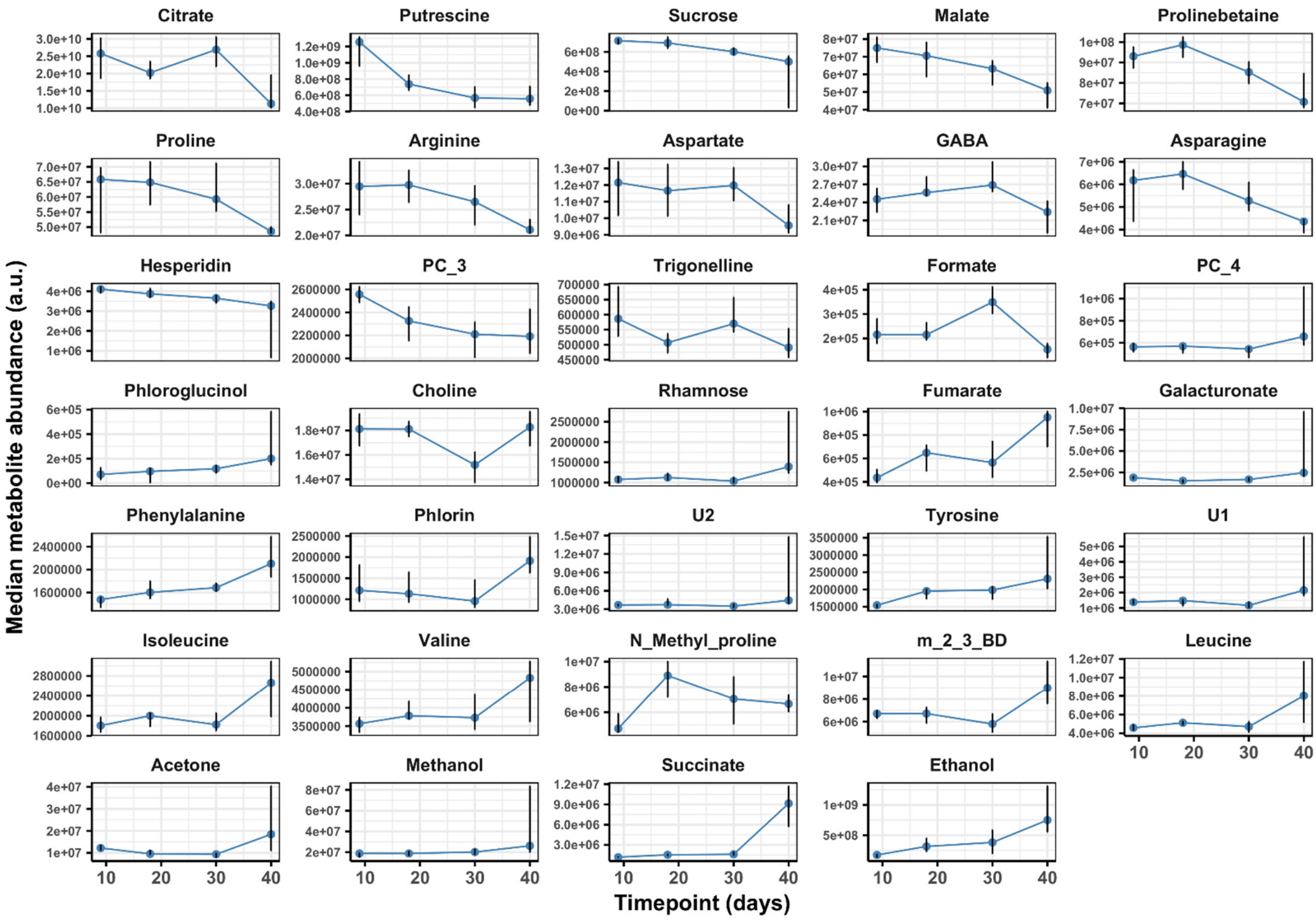

Figure S8. Cross-sectional metabolite trends (median ± IQR) for those metabolites whose change was significant at least in one pairwise MCCV-OPLS-DA model including D9 (n = 17), D18 (n = 8), D30 (n = 18), and D35 (n = 19) for the sensitivity analysis.

Table S1. ^{1}H and ^{13}C NMR peak assignments for identified metabolites in orange (*C. sinensis*) juice.

No.	Metabolite	δ ^{1}H	Multiplicity	δ $^{13}C^{a}$	Confirmation
1	Phenolic compound 1	0.73	s		1D, *J*-Res, TOCSY, STORM
		1.08	s		
		1.31	s		
		1.42	s		
		6.67	m		
		7.48	m		
		7.74	pss		
2	Isoleucine	0.94	t		1D, *J*-Res, COSY
		1.01	d		
		1.98	m		
3	Leucine	0.96	d		1D, *J*-Res, COSY
		0.97	d		
		1.73	m		
4	Valine	0.99	d		1D, *J*-Res, COSY
		1.04	d		
		2.27	m		
5	*meso*-2,3-Butanediol	1.14	d	19.66 (73.7)	1D, *J*-Res, COSY, TOCSY, HSQC, HMBC
		3.73	m		
6	U1	1.14	m		1D, STORM
		6.14	m		
7	Ethanol	1.19	t	19.7	1D, *J*-Res, COSY, TOCSY, HSQC, HMBC
		3.67	q	60.4	
8	Ethyl glucoside	1.24	t	17.16 (68.98)	1D, *J*-Res, COSY, TOCSY, HSQC, HMBC, STORM
		3.26	m		
		3.49	m		
		3.74	m		
		3.96	m		
		4.48	d		

9	Rhamnose	1.3	d		1D, STORM
		1.33	d		
10	Lactate	1.33	d	23.1	1D, *J*-Res, COSY, TOCSY, HSQC, HMBC
		4.13	q	70.8 (185.1)	
11	Alanine	1.48	d	19.09	1D, *J*-Res, COSY, TOCSY, HSQC, HMBC
		3.78	q		
12	U2	1.55	m		1D, STORM
		1.7	m		
		1.85	m		
13	Arginine	1.66	m	26.71	1D, *J*-Res, COSY, TOCSY, HSQC
		1.74	m	26.71	
		1.91	m	30.36	
		3.26	t		
		3.78	t		
14	Putrescine	1.78	m	26.8	1D, *J*-Res, COSY, TOCSY, HSQC, HMBC, STORM
		3.06	m	41.8	
15	4-Aminobutyrate	1.91	pent	26.15	1D, *J*-Res, COSY, TOCSY, HSQC, HMBC, STORM
	(GABA)	2.3	t	37.6	
		3.02	t	41.9 (184.35)	
16	Proline	1.98	m	26.7	1D, *J*-Res, COSY, TOCSY, HSQC, HMBC, STORM
		2	artefact		
		2.03	m		
		2.07	m	31.8	
		2.35	m	31.8	
		3.35	m		
		3.39	artefact		
		3.42	m		
		4.14	dd	64.3 (177.2)	
17	Prolinebetaine	2.16	m	21.35	1D, *J*-Res, COSY, TOCSY, HSQC, HMBC, STORM
		2.18	m	21.35	
		2.29	m	27.93	
		2.5	m	27.93	

	3.11	s	48.7 (69.9, 79.14)	
	3.3	s	54.8 (69.9, 79.14)	
	3.55	m	69.9	
	3.72	m	69.9	
	4.08	dd	79.14	
18	Acetone	2.24	s	33 (218.31)	1D, *J*-Res, HSQC, HMBC
19	Malate	2.39	dd	45.3 (182.54)	1D, *J*-Res, COSY, TOCSY, HSQC, HMBC
		2.68	dd	45.3	
		4.31	dd	73.2	
20	Succinate	2.42	s	36.6	1D, *J*-Res, HSQC
21	Citrate	2.55	d	48.3	1D, *J*-Res, COSY, TOCSY, HSQC, HMBC
		2.62	artefact		
		2.68	d	48.3 (78.03, 181.81, 184.43)	
22	Aspartate	2.7	dd		1D, *J*-Res, COSY, TOCSY
		2.76	artefact		
		2.81	dd		
		3.9	dd		
23	Asparagine	2.89	dd		1D, *J*-Res, COSY
		2.92	artefact		
		2.96	dd		
		4.01	dd		
24	*N*-methyl-Proline	2,94	s	43.62 (59.21, 73.44)	1D, *J*-Res, HSQC, HMBC
		3.16	m		
25	Choline	3.2	s	56.57 (70.26)	1D, *J*-Res, HSQC, HMBC
26	β-Glucose	3.26	dd	76.9	1D, *J*-Res, COSY, TOCSY, HSQC, HMBC, STORM
		3.41	dd	72.58	
		3.44	artefact		
		3.46	artefact		
		3.47	m	78.5	
		3.5	pst	78.64	
		3.73	m	63.64	

		3.91	dd	63.64	
		4.66	d	98.64	
27	Methanol	3.36	s	51.8	1D, *J*-Res, HSQC
28	α-glucose	3.42	dd	72.4	1D, *J*-Res, COSY, TOCSY, HSQC, HMBC, STORM
		3.55	dd	74.26	
		3.72	dd	75.71	
		3.77	m	63.49	
		3.82	artefact		
		3.83	m	63.49	
		3.85	m	75.1	
		5.25	d	94.7	
29	Sucrose	3.48	t	72.2	1D, *J*-Res, COSY, TOCSY, HSQC, HMBC, STORM
		3.57	dd	74.2	
		3.69	m	64.2	
		3.77	dd	75.5	
		3.83	m, 2x	63.2, 65.1	
		3.85	m	75.3	
		3.86	artefact		
		3.89	m	84.1	
		4.06	t	77.05	
		4.23	d	79.42	
		5.41	d	94.97 (106.52)	
30	Fructose	3.56	d	65.67 (104.43)	1D, *J*-Res, COSY, TOCSY, HSQC, HMBC, STORM
		3.57	d	66.91 (101)	
		3.59	artefact		
		3.61	d	65.67 (104.43)	
		3.67	m	65.8 (106.57)	
		3.69	m	65.27	
		3.71	m	66.13	
		3.73	d	66.74 (101)	
		3.81	d	70.41 (101)	
		3.86	artefact		

		3.91	dd	72.56	
		4.01	m	72.13	
		4.03	m	66.07 (101)	
		4.12	m	77.71	
31	Galacturonate	4.29	m		1D, *J*-Res, STORM
		4.42	psd		
		4.59	d		
		5.31	d		
32	Hesperidin	5.55	d		1D, *J*-Res, STORM
		6.28	m		
		7.09	m		
33	Phloroglucinol	6.02	s		1D, STORM
34	Phlorin[c]	6.19	pst		1D, *J*-Res, TOCSY, STORM
		6.23	psd		
35	Fumarate	6.53	s		1D, *J*-Res
36	Tyrosine	6.91	d[b]		1D, *J*-Res, COSY, TOCSY, STORM
		7.2	d[b]		
37	Phenolic compound 2	6.96	d[b]		1D, *J*-Res, COSY, TOCSY
		7.35	d[b]		
38	Phenolic compound 3	6.99	d[b]		1D, *J*-Res, COSY, TOCSY
		7.45	d[b]		
39	Phenylalanine	7.34	psd		1D, *J*-Res, COSY, TOCSY, STORM
		7.38	pst		
		7.44	pst		
40	Phenolic compound 4	7.59	m		1D, STORM
		8.16	m		
41	Formate	8.46	s		1D, *J*-Res
42	Trigonelline	8.85	m		1D, *J*-Res
		9.13	s		

[a]$\delta^{13}C$ HSQC (HMBC). [b]AA'BB'. [c]Phloroglucinol 1-O-β-D-glucoside, which is the phenolic phlorin rather than the photosynthesis-related compound. Key: s = singlet, d = doublet, t = triplet, q = quartet, pent = pentet, m = multiplet, dd = doublet of doublets, pss = pseudosinglet, psd = pseudodoublet, pst = pseudotriplet. Ui, unknown metabolites.

Table S2. Metabolites observed in orange (*C. sinensis*) whose changes in abundance were significantly associated with the differences observed across all timepoints.

Model	R^2Y[a]	Q^2Y[b]	Δ[c]	Metabolite	Association[d]
D9+D18 vs D30	0.76	0.63	0.13	Putrescine	↓
				Acetone	↓
				Malate	↓
				Succinate	↑
				Choline	↓
				Sucrose	↓
				Hesperidin	↓
				Phloroglucinol	↑
				Tyrosine	↑
				PC_3	↓
				Phenylalanine	↑
				Formate	↑
D9+D18 vs D35	0.68	0.63	0.05	Isoleucine	↑
				Leucine	↑
				Valine	↑
				m-2,3-BD	↑
				U1	↑
				Ethanol	↑
				Rhamnose	↑
				U2	↑
				Arginine	↓
				Putrescine	↓
				GABA	↓
				Proline	↓
				Prolinebetaine	↓
				Malate	↓
				Succinate	↑
				Citrate	↓

				Aspartate	↓
				Asparagine	↓
				Methanol	↑
				Sucrose	↓
				Galacturonate	↑
				Hesperidin	↓
				Phloroglucinol	↑
				Phlorin	↑
				Fumarate	↑
				Tyrosine	↑
				PC_3	↓
				Phenylalanine	↑
				PC_4	↑
D30 vs D35	0.62	0.58	0.04	Isoleucine	↑
				Leucine	↑
				m-2,3-BD	↑
				U1	↑
				Ethanol	↑
				Rhamnose	↑
				U2	↑
				GABA	↓
				Proline	↓
				Malate	↓
				Succinate	↑
				Citrate	↓
				Aspartate	↓
				Asparagine	↓
				Methanol	↑
				Sucrose	↓
				Galacturonate	↑
				Hesperidin	↓
				Phloroglucinol	↑

				Phlorin	↑
				Fumarate	↑
				Tyrosine	↑
				Phenylalanine	↑
				PC_4	↑
				Formate	↓
				Trigonelline	↓
D35(rt vs fp)[e]	0.31	0.03	N/A	N/A	N/A

[a,b]Validation parameters of the corresponding MCCV-OPLS-DA model. [c]Difference between R^2Y and Q^2Y. [d]Sign of association: ↑Upregulation, ↓downregulation at the latest time. [e]rt, reduced turgor; fp, firm pericarp tissue. Key: GABA, 4-aminobutyrate; m-2,3-BD, *meso*-2,3-butanediol; PCs, phenolic compounds; Ui, unknown metabolites. Sample sizes: D9+D18 (n = 25), D30 (n = 18), D35 (n = 19; rt, n = 11; yellow, fp, n = 8).

Table S3. Metabolites observed in orange (*C. sinensis*) whose changes in abundance were significantly associated with the differences observed across all timepoints for sensitivity analysis.

**Model**	**R^2Y[a]**	**Q^2Y[b]**	**Δ[c]**	**Metabolite**	**Association[d]**
D9 vs D18	0.77	0.3	0.47	Leucine	↑
				Valine	↑
				Ethanol	↑
				Putrescine	↓
				N-methyl-Proline	↑
				Tyrosine	↑
				Phenylalanine	↑
				Trigonelline	↓
D9 vs D30	0.87	0.79	0.08	Ethanol	↑
				Putrescine	↓
				Acetone	↓
				Malate	↓
				Succinate	↑
				N-methyl-Proline	↑
				Choline	↓
				Sucrose	↓
				Hesperidin	↓
				Fumarate	↑
				Tyrosine	↑
				PC_3	↓
				Phenylalanine	↑
				Formate	↑
D9 vs D35	0.7	0.66	0.04	Isoleucine	↑
				Leucine	↑
				Valine	↑
				m-2,3-BD	↑
				U1	↑
				Ethanol	↑
				Rhamnose	↑

				U2	↑
				Arginine	↓
				Putrescine	↓
				Prolinebetaine	↓
				Malate	↓
				Succinate	↑
				Citrate	↓
				Aspartate	↓
				Asparagine	↓
				N-methyl-Proline	↑
				Methanol	↑
				Sucrose	↓
				Galacturonate	↑
				Hesperidin	↓
				Phloroglucinol	↑
				Phlorin	↑
				Fumarate	↑
				Tyrosine	↑
				PC_3	↓
				Phenylalanine	↑
				PC_4	↑
				Trigonelline	↓
D18 vs D30	0.68	0.36	0.32	Choline	↓
				Sucrose	↓
				Formate	↑
D18 vs D35	0.63	0.55	0.08	Isoleucine	↑
				Leucine	↑
				m-2,3-BD	↑
				U1	↑
				Ethanol	↑
				Rhamnose	↑
				Arginine	↓

GABA	↓
Proline	↓
Prolinebetaine	↓
Malate	↓
Succinate	↑
Asparagine	↓
Methanol	↑
Sucrose	↓
Galacturonate	↑
Hesperidin	↓
Phloroglucinol	↑
Phlorin	↑
Fumarate	↑
Tyrosine	↑
Phenylalanine	↑
PC_4	↑

[a,b]Validation parameters of the corresponding MCCV-OPLS-DA model. [c]Difference between R^2Y and Q^2Y. [d]Sign of association: ↑Upregulation, ↓downregulation at the latest time. Key: GABA, 4-aminobutyrate; m-2,3-BD, *meso*-2,3-butanediol; PCs, phenolic compounds; Ui, unknown metabolites. Sample sizes: D9 (n = 17), D18 (n = 8), D30 (n = 18), D35 (n = 19).
